# The Influence of Introducing Free Vaccination against *Streptococcus pneumoniae* on the Uptake of Recommended Vaccination in Poland

**DOI:** 10.3390/vaccines11121838

**Published:** 2023-12-11

**Authors:** Wojciech Malchrzak, Mateusz Babicki, Dagmara Pokorna-Kałwak, Agnieszka Mastalerz-Migas

**Affiliations:** Department of Family Medicine, Faculty of Medicine, Wroclaw Medical University, 50-367 Wrocław, Poland; ma.babicki@gmail.com (M.B.); daga_kalwak@tlen.pl (D.P.-K.); agnieszka.migas@gmail.com (A.M.-M.)

**Keywords:** pneumococcal vaccine, vaccination program, voluntary vaccination

## Abstract

Since 2017, pneumococcal vaccination has evolved from a recommended chargeable vaccination to a mandatory, and therefore free, vaccination for all children. While a 10-valent vaccine is commonly used, parents have the option to use a 13-valent vaccine for a fee. This study aimed to investigate whether and how the introduction of free pneumococcal vaccination affected the uptake of recommended vaccination and to assess the association of chargeable pneumococcal vaccination with recommended vaccination. Data from 1595 vaccination record cards kept by six primary care clinics in urban and rural areas of Poland were collected and analyzed for children born between 2015 and 2018. Belonging to the clinic and the year of birth were the only inclusion criteria. Following the introduction of free universal pneumococcal vaccination, more children were vaccinated with the recommended vaccination (61.2% vs. 66.6%, *p* = 0.026). The most significant change was in vaccination against rotavirus (48.5% vs. 54.4%, *p* = 0.018) and against meningococcal B bacteria (4.8% vs. 17.0%, *p* < 0.001). Children who received chargeable pneumococcal vaccination were also significantly more likely to be vaccinated with recommended vaccines (54.6% vs. 75.9%, *p* < 0.001). In particular, this was the case for multivalent vaccinations—against rotavirus, chickenpox, and meningococcal C bacteria. Reducing the impact of the economic factor, for example, by introducing free vaccinations, should have a positive impact on the uptake of other recommended vaccinations.

## 1. Introduction

In Poland, the Preventive Vaccination Program (PVP) consists of mandatory and recommended vaccinations. Some compulsory vaccinations apply to all children, while others are dedicated exclusively to specific risk groups. All compulsory vaccinations are financed by the state budget. The recommended vaccination, on the other hand, is at the discretion of the parent, who bears the full cost [1,2].

Over the years, the PVP has expanded to include further compulsory vaccinations. One of these is the vaccination against *Streptococcus pneumoniae*, which, until the end of 2016, was only mandatory for a small group of patients with specific risk factors [3]. In 2017, this vaccination became mandatory for all children born after 1 January 2017. The same was true for the rotavirus (RV) vaccination, which was a recommended vaccination until 2021, and it has been on the mandatory vaccination list since 2021. According to the 2023 PVP in Poland, mandatory vaccinations include vaccination against tuberculosis (BCG), hepatitis B virus (HBV), rotavirus (RV), diphtheria, tetanus, pertussis (DTwP, whole-cell pertussis component or DTaP, cell-free pertussis component), H. Influenzae type B (HiB), pneumococcus (PCV), poliomyelitis (IPV), and measles, mumps, and rubella (MMR). On the other hand, the recommended vaccinations possible in the first two years of life include vaccination against *Neisseria meningitidis* group B (MenB), *N. meningitidis* group C (MenC), or groups A, C, W135, and Y (MenACWY), chickenpox (VZV), tick-borne encephalitis (TBE), and hepatitis A (HAV) [4].

It is also possible to opt for paid multivalent vaccinations instead of the standard DTwP, IPV, HiB, and HBV vaccinations. Two conjugate vaccines are available: a five-valent (‘5in1’), DTaP + IPV + HiB, and a six-valent (“6-in-1”), DTaP + IPV + HiB + HBV [4]. The 5-in-1 formulations are publicly reimbursed for children with contraindications to the whole-cell pertussis vaccine and for all children born before 37 weeks gestation or with a birth weight of less than 2500 g. The 6-in-1 vaccination is not state-funded [4]. Smallpox vaccination is compulsory for immunocompromised children and those around them, as well as for children in social and therapeutic institutions and those attending nursery school [4]. Pneumococcal vaccination in the youngest children can be performed with two conjugated vaccines: 10-valent (PCV10) or 13-valent (PCV13). PCV10 is used for universal mandatory vaccination. Parents can opt for PCV13, but in that case, they have to cover its cost. The only exception is infants born before 27 weeks gestation, as PCV10 is not registered for use in this patient group. Selecting PCV13 extends protection to three additional serotypes. Due to the similarity of pneumococcal antigens, immunity is also produced against some serotypes not included in the vaccine. This also happens with other vaccinations. For example, the smallpox vaccine can prevent monkeypox [5].

Nowadays, immunization is widely available. Nevertheless, their acceptance level, which has gradually decreased in recent years, is an enormous challenge. The increasing anti-vaccination movements are setting an increasing number of people against vaccination [6,7,8]. Acceptance of vaccination depends on many different factors, such as knowledge about vaccination or trust in the health system [9,10]. Economic issues are also relevant, as the price of vaccination can be a real barrier to its uptake [11]. Many studies have shown that pneumococcal vaccination is effective in preventing the disease, and thus money spent on vaccination saves money spent on treatment. Cost-effectiveness has been demonstrated not only for the elderly but also specifically for children [12,13]. Factors related to the vaccination itself are also important, such as the effectiveness of the vaccine and the risk of side effects. Observations carried out in connection with vaccination against COVID-19 in developing countries allow us to conclude that greater compliance and a lower risk of side effects have a positive impact on the reception of the vaccine in society [14]. Introducing a vaccination into the mandatory vaccination calendar and thus making it free of charge increases vaccination coverage and also reduces the incidence of a particular infection. The factor that is eliminated in such a case is the financial barrier [15,16]. However, it is not clear how the introduction of one vaccination into the calendar affects the performance of other vaccinations, particularly those that are not listed as free of charge. Many studies show that compulsory vaccination has an overall positive effect on vaccination uptake [17]. However, some studies suggest that compulsory vaccination may lead to reduced trust in the health system and paradoxically worsen acceptance not only of compulsory but also of other vaccinations [18,19].

Therefore, this study aims to assess whether the introduction of universal, compulsory, free pneumococcal vaccination in Poland in 2017 affected the implementation of recommended vaccination in this group of children. In addition, it assesses whether parents choosing paid pneumococcal vaccination are opting for a recommended vaccination. To the best of the authors’ knowledge, no similar study has been carried out since the introduction of compulsory vaccination. Nor is analogous data available from other countries.

## 2. Materials and Methods

### 2.1. Study Design

To be able to process the material, we had to prepare a database containing information on pneumococcal vaccinations and recommended vaccinations. These data can be found on vaccination cards that are kept in the primary health care facilities to which the patient belongs. To further diversify the study population, we searched for both urban and rural facilities.

### 2.2. Obtaining Data

After obtaining consent from the managers of six primary health care facilities, the vaccination record cards of children born between 2015 and 2018 residing in Wrocław (a Polish city of more than 500,000 inhabitants, four facilities) and in two surrounding villages (one facility in each) were placed in the database and then analyzed. All the available vaccination record cards were analyzed; the only inclusion criteria were being born in the year 2015–2018 and being a patient of the clinic at the time of data collection.

The database contained patient data in the form of date of birth and sex without the possibility of identifying the patient. The database also included dates of pneumococcal vaccinations with the formulation used, as well as information on multivalent (5-in-1 and 6-in-1) vaccinations against rotavirus infection, chickenpox, meningococcal disease, tick-borne encephalitis, and hepatitis A.

### 2.3. Study Group Settings

The records were then subdivided based on the child’s year of birth. The first group included children born in 2015 and 2016, and the second in 2017 and 2018. The cut-off point was when free pneumococcal vaccination was introduced.

**Figure 1:**
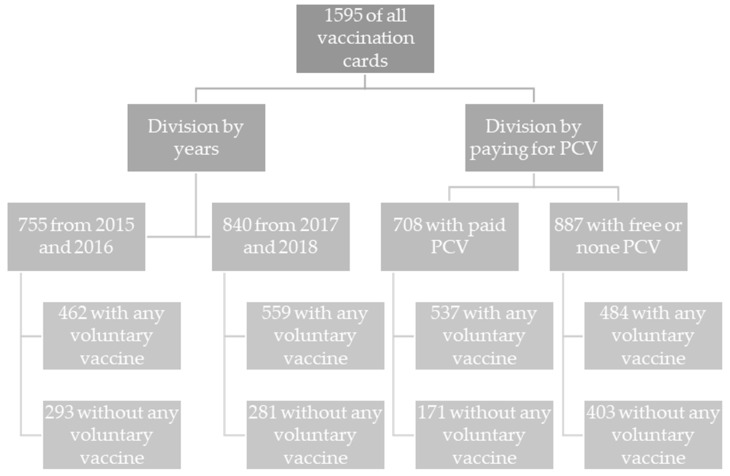
Flowchart of the breakdown of records into groups.

A breakdown was also made, taking into account the cost of pneumococcal vaccination. One group consisted of children who had received a chargeable pneumococcal vaccination and the other of those who had received either a free vaccine or none at all. Payments for pneumococcal vaccination are shown in Table 1, and the breakdown of records into groups is shown in in Figure 1.

### 2.4. Used Assumptions

Due to the different intervals between doses of recommended vaccination (if required), the administration of at least one dose of vaccination qualified the patient for the group of children who initiated recommended vaccination. The exception to this was the rotavirus vaccination, as there is a time limit to the completion of the vaccination schedule (32 weeks of age at the latest). In this way, it is possible to assess the willingness of parents to have a particular recommended vaccination.

Patients vaccinated against MenACWY were analyzed together in a group with patients vaccinated against MenC due to the epidemiological predominance of this meningococcal group.

The study was conducted in accordance with the Declaration of Helsinki and received a positive opinion from the Bioethics Committee of the Lower Silesian Medical Chamber. Opinion number 1/PNDR/2023.

### 2.5. Statistical Methods

The Chi-squared test was used to compare qualitative variables. In addition, a univariate logistic regression analysis was performed, where the dependent variable was the start of the recommended vaccination and the independent variable was the period before and after the introduction of compulsory pneumococcal vaccination in Poland. In the next step, a multivariate logistic regression analysis was performed while additionally taking into account the influence of the child’s age and place of residence. Statistical significance was assumed at the level of <0.05 Calculations were performed using Statistica 13 software from TIBCO Software Inc. (Palo Alto, CA, USA).

## 3. Results

### 3.1. Characteristics of the Study Group

The database yielded 1595 unique entries from vaccination record cards. Of these, 47.3 percent were children born in 2015–2016 and 52.7 percent were born in 2017–2018. Women constituted 52.2%. Clinics in urban areas accounted for 82.6% of the data. Full socio-demographic data are presented in Table 2.

### 3.2. Differences in Recommended Vaccination before and after Introducing Compulsory Pneumococcal Vaccination

Analysis of the results showed that after the introduction of free pneumococcal vaccination, immunization against rotavirus (48.5% vs. 54.4%, *p* = 0.018) and against meningococcal B bacteria (4.8% vs. 17.0%, *p* < 0.001) increased significantly. There was also a difference on the verge of statistical significance for tick-borne encephalitis vaccination (0.8% vs. 1.8%, *p* = 0.083). Furthermore, there was an increase in the proportion of children who received any recommended vaccination (61.2% vs. 66.6%, *p* = 0.026). Detailed data comparing the uptake of recommended vaccinations before and after the introduction of compulsory pneumococcal vaccination are shown in Table 3.

**Figure 2:**
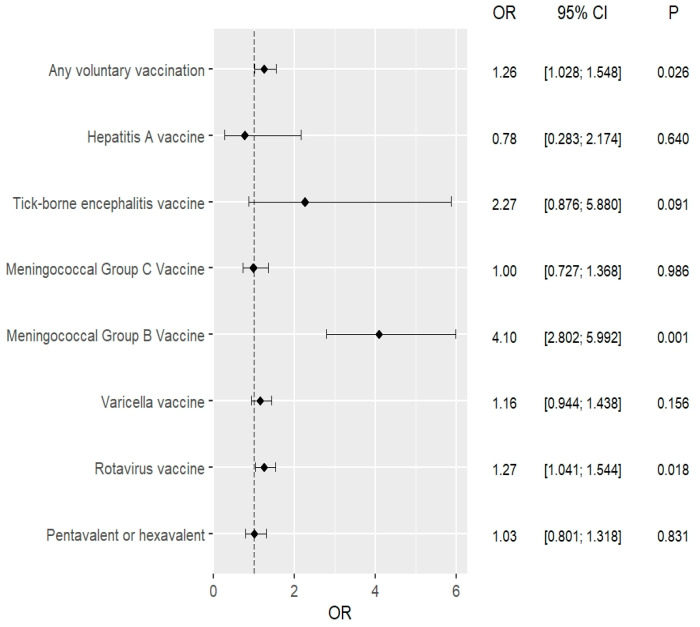
Univariate logistic regression assessing the impact of the introduction of compulsory pneumococcal vaccination in Poland on the performance of individual recommended vaccinations.

**Figure 3:**
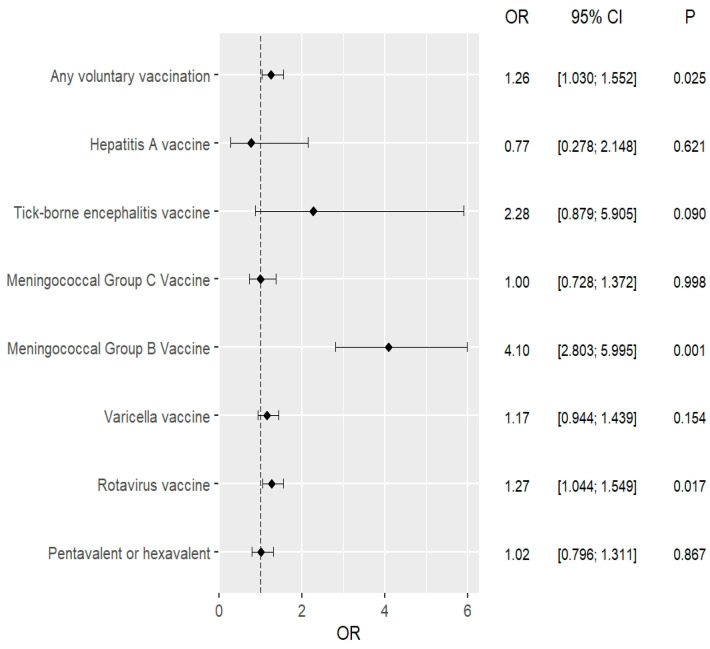
Multivariate logistic regression assessing the impact of the introduction of compulsory pneumococcal vaccination in Poland on the performance of individual recommended vaccinations, taking into account the age and place of residence of the child.

Univariate logistic regression analysis showed that after the introduction of compulsory pneumococcal vaccination, parents were significantly more likely to vaccinate their children against rotavirus and meningococcal B bacteria. Importantly, it also showed that the introduction of compulsory pneumococcal vaccination contributed to an increase in the uptake of recommended vaccination. These observations were also confirmed in a multivariate analysis that took into account the age and sex of the child. The exact results are shown in Table 4 and Figure 2 and Figure 3.

### 3.3. Differences in Recommended Vaccination Depenging on Vaccination with Chargeable PCV

Interestingly, comparing patients whose parents paid for the pneumococcal vaccination with those who had it free or were not vaccinated at all, it was shown that parents who paid the cost of the pneumococcal vaccination were also more likely to choose either the 5-in-1 or 6-in-1 conjugate vaccines against rotavirus infection, chickenpox, and meningococcal C bacteria (*p* < 0.001). These children were significantly more likely to receive any of the recommended vaccines (54.6% vs. 75.9%). The exact data has been collected in Table 5.

## 4. Discussion

The above study covered approx. 1.5 percent of all children born during the specified time period in the region—755 and 840 records in 2015–2016, and 2017–2018, respectively, out of 54,432 and 55,853 children [20,21,22,23]. It was shown that the introduction of compulsory pneumococcal vaccination may have influenced the frequency of recommended vaccination in the subgroup analyzed. First and foremost were vaccinations against meningococcal B bacteria and rotavirus. Notwithstanding the above change, it was observed that parents who opted for paid pneumococcal vaccination were more likely to have the recommended vaccination for their children. In this case, the difference was in conjugate vaccination against rotavirus, chickenpox, and meningococcal C bacteria.

Information on recommended vaccinations is provided to parents at clinic visits. Primarily during preventive appointments, such as patronage visits, health checks, and mandatory vaccination visits. The decision to have the recommended vaccination is entirely voluntary. There are notable benefits of vaccination. In the study group, 64% of the children had at least one recommended vaccination. This means that just over a third of parents stop at mandatory vaccinations only. This raises the question of why they do not want to protect their child against other diseases and what factors influence these decisions.

A Canadian systematic review found that vaccination uptake largely depends on confidence in vaccination and, more broadly, in the healthcare system. Mere knowledge about vaccinations is not a sufficient factor. Socio-economic factors are also linked to the willingness to vaccinate. Nevertheless, research indicates that a sufficiently high level of trust in healthcare professionals is able to offset the impact of these factors [24]. Similar conclusions were reached after a Swiss study in which parents’ doubts about vaccination were linked to a lack of trust in healthcare professionals [25]. Determining what is involved in reluctance to vaccinate is very difficult. A systematic review of studies from 2007 to 2012 noted many factors that may be associated with vaccine aversion but did not prove any of them to be universally associated [9]. Moreover, socioeconomic status: both low incomes and high incomes can account for reluctance to vaccinate. Similarly, a high level of education sometimes reinforces the decision to vaccinate and sometimes is a hindrance [26]. A 2015 study in Wrocław, the same city where the presented study was conducted, assessed immunization rates against pneumococcal infections, influenza, and pertussis. The pertussis vaccination was compulsory, and the other two were chargeable. Against pneumococcus, 36.8% of children were vaccinated, and against influenza—8%. Importantly, the authors pointed to the price of pneumococcal vaccination as a factor responsible for low vaccination rates. However, this still did not explain the even lower popularity of the relatively cheap flu vaccine [11]. In the presented study, following the introduction of free pneumococcal vaccination in children, recommended vaccinations were more frequently opted for. The situation was different with regard to vaccination against rotavirus, especially against meningococcal B bacteria. This study showed that the uptake of vaccination against these bacteria increased from 4.8% to 17%, representing a spectacular change. Studies comparing the determinants of pneumococcal and meningococcal vaccination indicate that they are largely similar [27]. Meningococcal B vaccine was placed on the market in 2012 [28] and became realistically available in Poland in 2014 [29]. It should be pointed out that there have been information campaigns on vaccinations in Poland, including against meningococci [30]. The campaign disseminated information about invasive meningococcal disease, its epidemiology, and, above all, vaccination [31]. Research confirms that disseminating information about vaccination improves vaccination uptake [32,33]. The primary objective is to address vaccination against a specific risk (in this case, the disease it protects against) and to dispel doubts that arise among patients [34]. Such campaigns have also been conducted with regard to other vaccinations. Approximately 5 years after the widespread meningococcal group B vaccination campaign, rotavirus vaccination campaigns were launched [35], but the effect was not as spectacular as for meningococci.

Fear of disease is undoubtedly a factor motivating vaccination—if a parent feels that the disease is not too dangerous, they will most probably be reluctant to vaccinate their child [36], or at least they will be willing to spend less on a potential chargeable vaccination [37]. Meanwhile, the 4CMenB vaccine, which is used in Poland, has very high efficacy, with a 79% to 100% reduction in the risk of invasive meningococcal disease compared to unvaccinated patients [38]. In Poland, theoretically, the vaccine should cover 86.6% of circulating group B meningococci. [39]. The effectiveness of the vaccine is also confirmed by the steadily decreasing incidence of invasive meningococcal disease. In 2021, it was 0.28/100,000, in 2017, 0.59, and in 2012, 0.63 [40,41,42]. For many years in Poland, the majority of cases of invasive meningococcal disease have been caused by type B bacteria [43]. Meningococcal vaccination is recommended for adults and children, with an emphasis on starting vaccination as early as possible, as meningococci are more dangerous the younger a child is [44]. The present study showed that the use of paid pneumococcal vaccination was associated with more frequent vaccination against meningococcal C infection and, more broadly, A, C, W135, and Y infection, as these types were part of the polyvalent vaccine used fairly frequently. In this case, too, vaccination shows effectiveness in preventing meningococcal disease [45].

Naturally, the limiting factor for vaccination is its cost. Sometimes the financial obstacle is insurmountable, and, despite the parents’ willingness, the child will not be vaccinated. Several studies have shown that the possibility of free vaccination increases the willingness to vaccinate [46]. Making pneumococcal vaccination free has removed the financial barrier. It is likely that some parents spent the money they would have spent on pneumococcal vaccination on other vaccinations. This study indirectly points to the importance of the financial factor. Parents who purchased the chargeable pneumococcal vaccine were also much more likely to opt for other recommended vaccinations (75.9% vs. 54.6%) compared to those parents who did not opt for the paid pneumococcal vaccine. A Polish study from 2013 found that a high cost of vaccines was associated with a five times lower chance of a child being vaccinated with them [47]. Moreover, if parents opt for chargeable vaccines, they will go for the multivalent variants of the mandatory vaccines first [48].

Among infants born in 2017–2018, more than half were vaccinated against rotavirus. This vaccine has a high efficacy level [49,50]. A factor that works in favor of this vaccination is the oral form of the vaccine. Parents do not have to overcome their child’s unwillingness to have an injection. Due to the efficacy and safety, as well as the cost-effectiveness of the vaccination, it was introduced into the Polish mandatory vaccination calendar in 2021 [51]. Of the other recommended vaccinations analyzed, no differences were observed among tick-borne encephalitis or hepatitis A vaccinations. These vaccinations are relatively unpopular, in addition to being possible after the age of one and being non-reimbursable. Only the chickenpox vaccination was received by about a third of children. Chickenpox vaccination, according to other studies, is accepted and even desired by parents, but cost remains the main obstacle to vaccination [52,53,54,55].

Despite the strength of the study, the authors are aware of its limitations. Undoubtedly, it should be mentioned that the study group is not representative of children in the Polish population born during this period. Nevertheless, it covers 1.5% of all births in the analyzed region. The study also did not assess parents’ opinions on vaccinations or their reasons for not opting for a particular vaccination. Juxtaposing such information with the data obtained from the vaccination record cards would allow the probable causes of the observed changes to be determined with more confidence. The authors believe that there is a need for further research on this topic, particularly on a larger sample of children, taking into account the representativeness of the group for the population of Polish children.

## 5. Conclusions

The introduction of compulsory pneumococcal vaccination has contributed to an increase in the frequency of opting for recommended vaccinations in the study group. A significant increase in the percentage of children vaccinated against meningococcal type B infections has been observed. This is probably related to the reduction of the economic barrier, which shows that one of the important aspects of the decision to vaccinate may be the economic factor. However, this hypothesis should be verified in further studies aimed at assessing the impact of economic factors on the willingness to vaccinate.

## Figures and Tables

**Table 1:** Payments for pneumococcal vaccination.

Vaccine	Until 2016	From 2017
PCV10 ^1^	Recommended; chargeable	Mandatory; free of charge
PCV13 ^2^	Recommended; chargeable	Recommended; chargeable

^1^ PCV10—10-valent conjugate pneumococcal vaccine. ^2^ PCV13—13-valent conjugate pneumococcal vaccine

**Table 2:** Characteristics of the study group.

Patient		Total Population *N* (%)	2015 and 2016 Age Group *N* (%)	2017 and 2018 Age Group *N* (%)	*p*
Sex	Male	763 (47.8)	387 (46.5)	445 (53.5)	0.525
	Female	832 (52.2)	368 (48.2)	395 (51.8)	
Place	Urban area	1318 (82.6)	630 (47.8)	688 (52.2)	0.417
	Rural area	277 (17.4)	125 (45.1)	152 (54.9)	

**Table 3:** Implementation of recommended vaccination before and after the introduction of compulsory pneumococcal vaccination. Statistically significant differences are shown in bold.

Recommended Vaccination		Total Population *N* (%)	2015 and 2016 Age Group *N* (%)	2017 and 2018 Age Group *N* (%)	*p*
5-in-1 or 6-in-1 conjugate vaccines	Yes	1288 (80.8)	608 (80.5)	680 (81.0)	0.831
	No	307 (19.2)	147 (19.5)	160 (19.0)	
Against rotavirus	Yes	823 (51.6)	366 (48.5)	457 (54.4)	0.018
	No	772 (48.4)	389 (51.5)	383 (45.6)	
Against chickenpox	Yes	516 (32.4)	231 (30.6)	285 (33.9)	0.156
	No	1079 (67.6)	524 (69.4)	555 (66.1)	
Against *N. meningitidis* B	Yes	179 (11.2)	36 (4.8)	143 (17.0)	<0.001
	No	1416 (88.8)	719 (95.2)	697 (83.0)	
Against *N. meningitidis* C	Yes	173 (10.8)	82 (10.9)	91 (10.8)	0.986
	No	1422 (89.2)	673 (89.1)	749 (89.2)	
Against tick-borne encephalitis	Yes	21 (1.3)	6 (0.8)	15 (1.8)	0.083
	No	1574 (98.7)	749 (99.2)	825 (98.2)	
Against hepatitis A	Yes	15 (0.9)	8 (1.1)	7 (0.8)	0.640
	No	1580 (99.1)	747 (98.9)	833 (99.2)	
Any recommended vaccinations	Yes	1021 (64.0)	462 (61.2)	559 (66.6)	0.026
	No	574 (36.0)	293 (38.8)	281 (33.5)	

**Table 4:** Univariate and multivariate logistic regression analysis.

Recommended Vaccinations	Univariate Analysis 2015–2016 vs. 2017–2018		Multivariate Analysis * 2015–2016 vs. 2017–2018	
	OR (95% CI)	*p*	OR (95% CI)	*p*
Conjugate (5-in-1 or 6-in-1) vaccines	1.028 (0.801–1.318)	0.831	1.022 (0.796–1.311)	0.867
Against rotavirus	1.268 (1.041–1.544)	0.018	1.271 (1.044–1.549)	0.017
Against chickenpox	1.165 (0.944–1.438)	0.156	1.166 (0.944–1.439)	0.154
Against *N. meningitidis* B	4.098 (2.802–5.992)	<0.001	4.099 (2.803–5.995)	<0.001
Against *N. meningitidis* C	0.997 (0.727–1.368)	0.986	1.000 (0.728–1.372)	0.998
Against tick-borne encephalitis	2.270 (0.876–5.880)	0.091	2.278 (0.879–5.905)	0.090
Against hepatitis A	0.784 (0.283–2.174)	0.640	0.773 (0.278–2.148)	0.621
Any recommended vaccinations	1.262 (1.028–1.548)	0.026	1.265 (1.030–1.552)	0.025

* age, place of residence.

**Table 5:** Children with recommended vaccination vaccinated with chargeable PCV vs. vaccinated with free PCV or not vaccinated against pneumococcus. Statistically significant differences are shown in bold.

Recommended Vaccination		Total Population *N* (%)	Free PCV or no PCV *N* (%)	Chargeable PVC *N* (%)	*p*
5-in-1 or 6-in-1 conjugate vaccines	Yes	1288 (80.8)	651 (73.4)	637 (90.0)	<0.001
	No	307 (19.2)	236 (26.6)	71 (10.0)	
Against rotavirus	Yes	823 (51.6)	376 (42.4)	447 (63.1)	<0.001
	No	772 (48.4)	511 (57.6)	261 (36.9)	
Against chickenpox	Yes	516 (32.4)	249 (28.1)	267 (37.7)	<0.001
	No	1079 (67.6)	638 (71.9)	441 (62.3)	
Against *N. meningitidis* B	Yes	179 (11.2)	97 (10.9)	82 (11.6)	0.685
	No	1416 (88.8)	790 (89.1)	626 (88.4)	
Against *N. meningitidis* C	Yes	173 (10.8)	63 (7.1)	110 (15.5)	<0.001
	No	1422 (89.2)	824 (92.9)	598 (84.5)	
Against tick-borne encephalitis	Yes	21 (1.3)	11 (1.2)	10 (1.4)	0.764
	No	1574 (98.7)	876 (98.8)	698 (98.6)	
Against hepatitis A	Yes	15 (0.9)	5 (0.6)	10 (1.4)	0.081
	No	1580 (99.1)	882 (99.4)	698 (98.6)	
Any recommended vaccinations	Yes	1021 (64.0)	484 (54.6)	537 (75.9)	<0.001
	No	574 (36.0)	403 (45.4)	171 (24.2)	

## Data Availability

The heads of the primary care facilities where the data were collected agreed only to the collective presentation of the analysis results without the possibility of publishing the full database.
